# Risk factors associated with multidrug-resistant pulmonary tuberculosis in patients over 15 years old: a retrospective case-control study

**DOI:** 10.3389/ftubr.2025.1608364

**Published:** 2025-07-18

**Authors:** Odete Bambi Receado, Paulo Ney Solary, Emanuel Catumbela, Carlos Alberto Pinto de Sousa

**Affiliations:** ^1^Department of Public Health, Faculty of Medicine, Agostinho Neto University, Luanda, Angola; ^2^Clínica Sagrada Esperança, Luanda, Angola; ^3^Department of Microbiology, Pathology and Diagnostic Methods, Faculty of Medicine, Agostinho Neto University, Luanda, Angola; ^4^Department of Public Health, Faculty of Medicine, Agostinho Neto University, Luanda, Angola

**Keywords:** multidrug-resistant pulmonary tuberculosis, risk factors, irregular treatment, Angola, epidemiology

## Abstract

**Introduction:**

Multidrug-Resistant Pulmonary Tuberculosis (MDR-TB) represents a serious public health problem, hindering disease control and increasing morbidity and mortality. This study aimed to identify risk factors associated with MDR-TB in patients over 15 years old at the Sanatorium Hospital of Luanda during the period 2015–2016.

**Methods:**

A retrospective case-control study (1:1) was conducted with 500 participants, including 250 cases (MDR-TB) and 250 controls (drug-sensitive TB). Data were obtained from clinical records and analyzed using logistic regression in Epi-Info 7.2.1.0 software, considering a significance level of *p* < 0.05.

**Results:**

MDR-TB was more prevalent in men (61.6%), in the age group 20–29 years (36.8%), and among employed individuals (73.2%). The associated risk factors were irregular medication adherence [odds ratio (OR) = 12.3; *p* < 0.001], previous TB treatment (OR = 9.0; *p* < 0.001), contact with an MDR-TB patient (OR = 4.1; *p* < 0.001), and lower educational level (OR = 2.1; *p* = 0.03).

**Conclusion:**

Irregular adherence to treatment and a history of previous TB were the main factors associated with MDR-TB, reinforcing the need for effective strategies to ensure treatment follow-up.

## Introduction

Multidrug-Resistant Pulmonary Tuberculosis (MDR-TB) represents a serious and challenging public health issue, with an increasing trend in various parts of the world. It poses a significant threat to Tuberculosis (TB) control programs (1). Rapid diagnosis, adherence to recommended therapeutic guidelines, epidemiological surveillance, and strict case monitoring are essential to preventing the expansion of resistance and the transmission of multidrug-resistant (MDR) strains (2).

The World Health Organization (WHO) defines MDR-TB as resistance to at least two essential ant tuberculosis drugs, specifically when *Mycobacterium tuberculosis* demonstrates *in vitro* resistance to both Isoniazid and Rifampicin simultaneously (3).

The development of resistance to tuberculosis drugs was first documented in the late 1940s. During the 1960s, strains of *Mycobacterium tuberculosis* resistant to Streptomycin and Isoniazid were identified—two of the main drugs used in the treatment of the disease before the introduction of Rifampicin (4).

In the 1980s, immunosuppression, particularly in infected elderly individuals, facilitated the reactivation of TB and contributed to the emergence of strains resistant to the Rifampicin-Isoniazid combination (4).

At the end of the 1980s and the early 1990s, a global alert was issued regarding MDR-TB following the identification of ~300 cases in eight hospitals and a prison in the United States of America (USA). It was only in the early 1990s that MDR-TB came to be globally recognized as a public health threat. The epidemic of the disease among individuals living with the Human Immunodeficiency Virus (HIV) in the USA and Europe further highlighted the severity of the problem (5).

Its publication triggered such an intense international outcry that, in 1993, the WHO declared TB a “global emergency,” characterizing it as a “worldwide public health crisis.” Until then, the disease had been neglected in several regions, leading to a loss of priority in its control efforts and the emergence of MDR-TB outbreaks, even in developed countries (6).

In 1994, the WHO, with the support of the International Union Against Tuberculosis and Lung Disease, established the Global Project on Anti-Tuberculosis Drug Resistance Surveillance. This initiative, commonly referred to as “the project,” was created to assess the magnitude of drug resistance and monitor its evolution over time. Today, it remains the largest and longest-standing antimicrobial resistance surveillance initiative in the world (7).

The objective of this study was to assess the risk factors associated with MDR-TB in patients over 15 years old at the Sanatorium Hospital of Luanda from 2015 to 2016.

This study aimed to identify risk factors associated with MDR-TB in patients over 15 years old at the Sanatorium Hospital of Luanda during the period 2015–2016.

## Methods

### Study design

A retrospective case-control study was conducted in a 1:1 ratio, unmatched.

### Study period

The study was carried out between January 2015 and December 2016.

### Study location

The study was conducted at the Sanatorium Hospital of Luanda, a tertiary-level healthcare facility and a national reference center for the treatment of tuberculosis (TB) and other respiratory diseases. Located in the Kilamba-Kiaxi municipality, Palanca urban district, the hospital covers an area of 21.3 hectares and has been operational since July 15, 1972. It serves an average of 200 patients per day, with a capacity of 250 beds, although ~293 patients are often hospitalized simultaneously. Additionally, more than 2,000 outpatients receive medical care. The hospital comprises various healthcare services, including consultation rooms, laboratories, pharmacies, radiology services, and a Voluntary Counseling and Testing Center. The medical team consists of 25 doctors (18 Angolans and seven Cubans), 215 nurses, and 298 support staff. Since 2013, the institution has been monitoring the first cases of drug-resistant tuberculosis.

### Study population

The study population consisted of patients with Multidrug-Resistant Pulmonary Tuberculosis (MDR-TB) and patients with drug-sensitive tuberculosis (DSTB) who were considered cured after treatment.

### Inclusion and exclusion criteria

The study included patients over 15 years old who were undergoing treatment and follow-up at the Sanatorium Hospital of Luanda and met the WHO definition of MDR-TB, as well as patients over 15 years old with pulmonary tuberculosis who were considered cured after treatment at the same hospital. The study excluded patients with extrapulmonary tuberculosis, and cases with incomplete clinical records regarding the study variables.

### Case and control definition

A case was defined as a patient with laboratory-confirmed MDR-TB who was undergoing treatment and follow-up at the Sanatorium Hospital of Luanda.

A control was defined as a patient with drug-sensitive tuberculosis (DSTB) who was considered cured after treatment at the same hospital.

### Sample size calculation

The sample size was calculated using the electronic calculator of Epi-Info software version 7.2.1.0, applying Kelsey's formula. The parameters considered included a 95% confidence interval (CI), 80% study power, 12% exposure in cases, and 5% exposure in controls. The final sample consisted of 500 participants, comprising 250 cases and 250 controls.

### Sampling method

A list of patients treated at the Sanatorium Hospital of Luanda during the study period (January 2015 to December 2016) was obtained. From this list, patients who met the inclusion criteria for cases (laboratory-confirmed MDR-TB) were identified. Clinical records were consecutively reviewed and included until the required sample size of 250 cases was reached. A parallel process was followed for controls (patients with drug-sensitive TB considered cured after treatment), with 250 controls also consecutively included from the hospital's records.

Data were primarily extracted from the patients' clinical records. In instances where examination results were missing from the individual records, supplementary information was retrieved directly from the hospital's imaging and laboratory services to ensure data completeness for the study variables.

### Data collection instrument

Data collection was conducted using a structured, closed-ended questionnaire, which was pre-tested at the Sanatorium Hospital of Luanda. The questionnaire included information on sociodemographic data, clinical history, laboratory tests, and risk factors, obtained from patient medical records and hospital registries.

### Data processing and analysis

The questionnaires were reviewed to ensure data integrity, and the sequence of completion was maintained throughout the process. Qualitative and quantitative data were categorized and analyzed using Epi-Info software version 7.2.1.0. The analysis included frequencies and proportions, central tendency and dispersion measures (median and interquartile range), significance tests (Chi-square test), and multivariate logistic regression analysis to express the strength of associations through odds ratio (OR) and 95% confidence intervals (CIs). Variables were considered statistically significant when the CI did not include 1 and *p* < 0.05.

### Definition of study variables

The dependent variable was Multidrug-Resistant Pulmonary Tuberculosis (MDR-TB), defined as patients with a positive laboratory result for resistance to both Isoniazid and Rifampicin. The independent variables included sociodemographic, clinical, and laboratory factors, extracted from medical records and hospital registries.

### Sociodemographic, clinical, and laboratory variables

Sociodemographic variables:

- Age: (< 20, 20–29, 30–39, 40–49, and >49 years)
- Sex: (male or female)
- Occupation: (employed or unemployed)
- Educational level: (below 12th grade or above 12th grade)
- Incarceration status: (yes or no)
- Homelessness: (yes or no).

Clinical variables:

- Number of previous treatments: (first-time, 1–2 times, or >2 times)
- History of TB/MDR-TB: (yes or no)
- Supervised treatment: (not supervised or supervised for 3–6 months)
- Time taken to seek medical care: (immediate—within 4 weeks—or delayed—after 4 weeks)
- Alcohol consumption: (yes or no)
- Tobacco use: (yes or no)
- Contact with a TB/MDR-TB patient: (yes or no)
- Family history of TB/MDR-TB: (yes or no)
- Irregular medication adherence: (yes or no)
- Primary resistance: (yes or no)
- Secondary resistance: (yes or no)
- HIV/AIDS infection: (yes or no).

### Ethical considerations

The study was submitted for review by the National Ethics Committee of the Ministry of Health of Angola. Data collection posed no physical risks to participants, as it was based solely on medical records and hospital registries. Permission to conduct the study was granted by the Directorate of the Sanatorium Hospital of Luanda. Data confidentiality was ensured throughout the study and will be maintained after its conclusion.

## Results

Regarding the sociodemographic characteristics of the overall study participants (*n* = 500), 292 (58.4%) were male and 208 (41.6%) were female (*p* = 0.17). The age distribution showed that 39 (7.8%) were aged < 20 years, 162 (32.4%) were 20–29 years, 143 (28.6%) were 30–39 years, 91 (18.2%) were 40–49 years, and 65 (13.0%) were >49 years, with a median age of 32 years (*p* = 0.8). Most participants, 358 (71.6%), were employees, while 142 (28.4%) were unemployed (*p* = 0.48). Concerning marital status, 286 (57.2%) had partners and 214 (42.8%) had no partners (*p* = 0.008). In terms of academic level, 458 (91.6%) had attained a level below the 12th grade, and 42 (8.4%) had a higher education level (*p* = 0.3).

Focusing on the characteristics of the Multidrug-Resistant Tuberculosis (MDR-TB) cases (*n* = 250), males predominated, accounting for 61.6% (154/250) of cases. The most affected age group was 20–29 years, representing 36.8% (92/250) of cases. Among occupations, employees constituted the largest proportion at 73.2% (183/250). For marital status, individuals with partners were the most affected, comprising 51.2% (128/250) of cases. Notably, 94.4% (236/250) of the MDR-TB patients had an academic level below the 12th grade (Table 1).

**Table 1:** Distribution of socio-demographic characteristics of study participants at the Luanda Sanatorium Hospital, 2015/2016.

**Variables**	**Case**		**Control**		**Total**		***p*-valor**
	***n*** **=** **250**	**%**	***n*** **=** **250**	**%**	***n*** **=** **500**	**%**	
Age (median and interquartile)	32 (25–42)					
Sex							
Male	154	61.6	138	55.2	292	58.4	0.17
Female	96	38.4	112	44.8	208	41.6	
Occupation							
Employees	183	73.2	175	70.0	358	71.6	0.48
Unemployed	67	26.8	75	30.0	142	28.4	
Marital status							
With partners	128	51.2	158	63.2	286	57.2	0.008
No partners	122	48.8	92	36.8	214	42.8	
Academic level							
Level below the 12th	236	94.4	222	88.8	458	91.6	0.03
Higher level	14	5.6	28	11.2	42	8.4	

Table 2 presents the bivariate statistical analysis of the possible risk factors associated with the occurrence of MDR-TB in patients treated at the Luanda Sanatorium Hospital during the period 2015–2016. Factors demonstrating a statistically significant association with MDR-TB included: irregular medication intake [odds ratio (OR) = 12.3; 95% CI = (7.6–19.7); *p* < 0.001], having contact with a TB/MDR-TB patient [OR = 4.1; 95% CI = (2.7–6.3); *p* < 0.001], and having a family history of MDR-TB [OR = 1.5; 95% CI = (1.0–2.2); *p* = 0.02]. Treatment non-supervision [OR = 2.1; 95% CI = (1.4–3.0); *p* < 0.001] and delayed time in seeking a health unit [OR = 1.4; 95% CI = (1.0–2.1); *p* = 0.03] also showed a relevant association. Other factors significantly associated with TB were: having a prior history of MDR-TB treatment [OR = 9.0; 95% CI = (5.9–14); *p* < 0.001], tobacco use [OR = 2.2; 95% CI = (1.6–3.3); *p* < 0.001], lower academic level [OR = 2.1; 95% CI = (1.0–4.1); *p* = 0.03], not having a partner [OR = 1.6; 95% CI = (1.6–2.3); *p* = 0.008], and being HIV positive [OR = 2.1; 95% CI = (1.1–4.0); *p* = 0.01]. Irregularity in medication intake exhibited the strongest association with MDR-TB, suggesting that inadequate adherence to treatment significantly increases the risk of developing the disease.

**Table 2:** Bivariate statistical analysis of possible risk factors for contracting MDR-TB at the Luanda Sanatorium Hospital, 2015/2016.

**Variables**	**OR**	**95% CI**	***p*-valor**
The irregularity in taking the medicines	12.3	[7.6–19.7]	0.000
Having contact with a carrier with MDR-TB	4.1	[2.7–6.3]	0.000
Having a family history of MDR-TB	1.5	[1.0–2.2]	0.02
Non-supervision of the treatment	2.1	[1.4–3.0]	0.000
Time taken to seek medical care	1.4	[1.0–2.1]	0.03
Have prior treatment of MDR-TBTB	9.0	[5.9–14]	0.000
Tobacco use	2.2	[1.6–3.3]	0.000
Lower academic level	2.1	[1.0–4.1]	0.03
Not having a partner	1.6	[1.6–2.3]	0.008
Being HIV positive	2.1	[1.1–4.0]	0.01

Furthermore, when using the multivariate analysis of the variables, as shown in Table 3, and employing a logistic regression model to rule out possible confounding factors, the final adjusted model demonstrated statistically significant associations for the following factors: irregularity in the taking of medications [OR = 51.8; 95% CI = (6.4–416); *p* = 0.0002] and having had previous treatment for MDR-TB [OR= 14.4; 95% CI = (1.6–126); *p* = 0.01].

**Table 3:** Multivariate statistical analysis of risk factors for contracting MDR-TB at the Luanda Sanatorium Hospital, 2015/2016.

**Variables**	**Bivariate analysis**		**Multivariate analysis**		
	**OR**	**IC-95%**	**Hour**	**95% CI**	***p*** **-value**
Irregularity in the taking of DRUGS	12.3	7.6–19.7	51.8	6.4–416	0.0002
Have previous treatment of TBMDR/TB T	9.0	5.9–14	14.4	1.6–126	0.01

## Discussion

Our study revealed a higher prevalence of multidrug-resistant tuberculosis (MDR-TB) among males (62%). This finding aligns with observations from the 2015 Annual Report/Angola (PNCT) (8) and several studies by Marques et al. (9–13), which also reported a higher incidence of tuberculosis in men. This trend may be associated with men's potentially lower engagement with healthcare services and increased exposure to TB risk factors. In the Angolan context, men often prioritize work and may delay seeking medical attention due to factors such as long waiting times, as we noted. This is further supported by Sebastião et al.'s (17) study in Luanda, Angola, which identified male gender as a significant risk factor for drug-resistant TB.

The highest prevalence of MDR-TB was observed in the 20–29 year age group. This may be attributed to Angola's predominantly young and economically active population (8), coupled with potential disease denial and fear of stigma leading to delays in diagnosis and treatment. These findings are consistent with Marques et al. (9) and Pires et al. (12), who also reported higher TB prevalence in similarly aged populations, often engaged in work environments with increased risk of TB transmission.

Interestingly, while unemployed individuals were initially expected to be more vulnerable, employed workers also exhibited a high prevalence of MDR-TB. This suggests that occupational hazards and working environments may play a significant role in TB transmission within this population.

The association of having a partner with a higher prevalence of TB, potentially due to increased work intensity to support families, presents a notable observation. Conversely, the initial expectation of higher prevalence in single individuals highlights the complex interplay of social factors and TB risk.

Lower educational attainment was identified as a key determinant of health and a potential risk factor for MDR-TB, likely hindering the understanding and adherence to complex treatment regimens. This underscores the importance of targeted educational interventions.

Irregular medication adherence emerged as the most significant risk factor for MDR-TB. This is corroborated by Barroso et al.'s study (14), which reported a substantial odds ratio (OR = 5.14, 95% CI = 2.37–11.16) for treatment irregularity being associated with MDR-TB. Factors such as the long treatment duration (18–24 months), high pill burden (more than 12 tablets per day), adverse effects, lack of family support, financial difficulties, social discrimination, and limited drug availability likely contribute to this poor adherence. Vita et al.'s (18) study on loss to follow-up during TB treatment in Luanda highlights factors that can lead to treatment interruption, potentially contributing to drug resistance.

The high proportion of secondary resistance (86%) compared to primary resistance (14%) strongly indicates that acquired resistance due to inadequate treatment, interruption, or irregular adherence is the dominant pathway to MDR-TB in our study population. This aligns with findings from Namburete et al. (15), Vieira et al. (16), and the World Health Organization (WHO) (3). This contrasts with findings from developed countries, where primary resistance may be more prevalent due to institutional transmission and migration (4). The recent study by Francisco et al. (19) in a community hospital in Luanda further emphasizes the ongoing challenge of drug-resistant TB in this setting. Previous exposure to anti-tuberculosis drugs and potentially incomplete or inadequate prior treatment courses likely contribute significantly to this high rate of secondary resistance by exerting selection pressure for drug-resistant strains.

While our study did not directly focus on loss to follow-up, Augusto et al.'s (20) study in Angola provides valuable insights into factors affecting treatment adherence and patient retention, such as socio-economic challenges and healthcare access issues. These factors could indirectly contribute to the development of drug resistance due to treatment interruption. Furthermore, Segagni Lusignani et al.'s (21) study on delays in TB diagnosis in Luanda suggests that prolonged infectiousness due to delayed diagnosis could potentially influence the transmission of drug-resistant strains.

### Limitations

This study benefits from a robust case-control design, allowing for the identification of key risk factors for MDR-TB within the Angolan context. The significant association found between irregular treatment adherence and MDR-TB, further highlighted through multivariate analysis, underscores a critical area for intervention. Furthermore, the study's clear methodology and statistical rigor strengthen the reliability of these findings. However, inherent limitations of its retrospective nature, relying on existing medical records, must be acknowledged. The single-center design may also limit the generalizability of the results to other settings within Angola. Additionally, while several socio-demographic and clinical factors were examined, the depth of exploration into certain aspects, such as the specifics of previous TB treatments and detailed socio-economic determinants, was limited.

We acknowledge a potential selection bias inherent in the definition of our study groups. While cases were defined as patients with laboratory-confirmed multidrug-resistant tuberculosis (MDR-TB) undergoing treatment and follow-up, controls were characterized as patients with drug-sensitive tuberculosis (DSTB) who were considered cured after treatment. This distinction in the participants' disease stages and treatment pathways may have introduced fundamental differences between the groups, beyond the direct risk factors for MDR-TB.

Specifically, selecting controls who had already successfully completed treatment and been deemed cured might under-represent the true heterogeneity of DSTB patients, excluding those who faced adherence challenges or less favorable outcomes. Such an approach could, theoretically, amplify the magnitude of observed associations between risk factors and MDR-TB, as the controls may represent a population with a greater propensity for therapeutic success.

Although this approach was dictated by data availability and the retrospective design, future studies would benefit from selecting controls at the initial diagnostic stage for a more equitable comparison with cases, or from an analysis that explores the influence of treatment status at the time of inclusion. Despite this potential bias, our findings regarding irregular adherence and a history of previous treatment remain robust and consistent with the literature, underscoring their importance regardless of the specific group definitions.

It is important also to acknowledge a temporal limitation of the present study, given that data collection occurred between 2015 and 2016. While our results reflect the epidemiological situation of multidrug-resistant tuberculosis during that period, we understand that a decade has passed until publication. This gap may directly influence the currency and immediate applicability of some conclusions to the epidemiological scenario of 2025. Nevertheless, the identified risk factors, such as irregular treatment adherence and previous TB treatment, are persistent determinants in the emergence of MDR-TB, underscoring the continuous relevance of these findings for disease control strategies. Future investigations, with more recent data, will be crucial to monitor the evolution of these factors and the effectiveness of interventions.

Finally, we acknowledge that our study did not collect detailed data on other forms of immunosuppression beyond HIV/AIDS, nor did it include information on CD4 cell counts for HIV-positive patients. While HIV infection was identified as a risk factor, the absence of more granular data on immune status may limit a deeper understanding of its precise role in MDR-TB development within our cohort. Future research should aim to capture these additional clinical parameters to provide a more comprehensive assessment of immune-related risk factors.

## Conclusion

Multidrug-resistant pulmonary tuberculosis (MDR-TB) remains a significant public health concern, with a higher prevalence observed among males and individuals aged 20–29 years. Our findings suggest that occupational factors may contribute to the risk, as employed workers also exhibited a high prevalence. Low educational attainment appears to be associated with the disease, likely impacting the understanding and adherence to treatment regimens. Irregular medication adherence emerged as the most potent risk factor for MDR-TB, influenced by the extended treatment duration, substantial medication burden, adverse effects, insufficient direct supervision, and financial constraints. Furthermore, the predominance of acquired resistance underscores the critical need for robust Directly Observed Treatment (DOT) programmes, improved access to a consistent supply of medications, and comprehensive educational initiatives aimed at enhancing treatment adherence.

## Data Availability

The raw data supporting the conclusions of this article will be made available by the authors, without undue reservation.
